# Mukbang Addiction and Emotional Eating Among University Students in Saudi Arabia: A Cross-Sectional Study

**DOI:** 10.3390/nu18152554

**Published:** 2026-08-05

**Authors:** Fatimah Alenazi, Hend F. Alharbi

**Affiliations:** 1 Department of Public Health, College of Applied Medical Sciences, Qassim University, Buraydah 51452, Saudi Arabia; f.alenazi@qu.edu.sa; 2 Abdullah Al-Othaim Diabetes Center, Medical City, Qassim University, Buraydah 52571, Saudi Arabia; 3 Department of Food Science and Human Nutrition, College of Agriculture and Food, Qassim University, Buraydah 51452, Saudi Arabia; 4 Department of Clinical Nutrition, Medical City, Qassim University, Buraydah 52571, Saudi Arabia

**Keywords:** Mukbang, emotional eating, public health, students, nutrition, diet

## Abstract

**Background/Objectives**: Mukbang videos have become increasingly popular and may be associated with eating behaviors, particularly among university students who are more susceptible to stress-related eating and social media use. Evidence on the relationship between Mukbang addiction and emotional eating is limited. This study investigated the association between Mukbang addiction and emotional eating among university students in Saudi Arabia. **Methods**: A cross-sectional study was conducted between November 2025 and April 2026 using an online questionnaire. A total of 551 students completed the survey. Data were analyzed using descriptive statistics, chi-square tests, and multiple linear regression. **Results**: Emotional Eating Questionnaire (EEQ) and Mukbang Addiction Scale (MAS) scores did not differ significantly by age, gender, education, or income. EEQ scores differed significantly by marital status (*p* = 0.025) and occupation (*p* = 0.037), while MAS did not. Both EEQ and MAS were significantly associated with BMI (*p* < 0.05). Descriptive analysis showed that participants with high emotional eating levels had a greater proportion of high Mukbang addiction (55%) compared with low Mukbang addiction (45%). However, multiple linear regression analysis found no significant association between EEQ and MAS scores (B = 0.275, *p* = 0.249). Additionally, MAS scores were not significantly associated with any demographic characteristics. **Conclusions**: BMI was significantly linked to both emotional eating and Mukbang addiction. Longitudinal studies are needed to clarify these relationships and inform interventions promoting healthier eating behaviors among university students.

## 1. Introduction

Online food-related content, such as Mukbang, has grown globally. The term Mukbang is a South Korean word that combines Mukja, meaning “to eat,” and Bangsong, meaning “broadcasting,” and refers to live or recorded videos in which a person consumes a large quantity of food [1]. These videos became popular after being published on well-known social media and video platforms such as YouTube and TikTok, and they have millions of viewers [2,3]. The appeal of Mukbang may extend beyond entertainment, potentially involving social and emotional elements that influence viewers’ emotions and behaviors [4,5,6].

Although there is no direct evidence explaining the relationship between watching Mukbang videos and emotional eating, several studies have proposed psychological and behavioral mechanisms. Mukbang videos present highly appealing food cues. Close-up images of large portions, amplified eating sounds, and the host’s visible enjoyment of food can stimulate appetite and cravings in ways similar to real-life food exposure. Evidence suggests that such cues may trigger eating even in the absence of physiological hunger [7,8,9]. Food cue reactivity and craving function as conditioned responses that recruit reward-related pathways and promote intake independently of energy need. A meta-analysis of 45 studies found that cue reactivity and craving prospectively predicted eating and weight gain, and that visual cues such as pictures and videos produced effects comparable in magnitude to exposure to real food, indicating that screen-based food content of the kind featured in Mukbang can act as a potent conditioned stimulus [10]. Mukbang creators also frequently consume large quantities of food, appearing comfortable and free from any negative effects. Repeated exposure to this behavior may change viewers’ perceptions of appropriate portion sizes and encourage greater food consumption over time [3,11]. Many viewers report watching Mukbang videos when experiencing boredom, loneliness, or stress, which are well-recognized triggers of emotional eating. Consequently, Mukbang viewing and emotional eating may represent related strategies for coping with negative emotions [8,12]. Mukbang also provides viewers with a sense of connection. Hosts often engage directly with viewers and create the impression of sharing a meal together. This social connection may be particularly appealing to individuals experiencing loneliness, strengthening the association between food and emotional comfort [5,6]. Mukbang viewing may therefore influence eating behavior through food cue exposure, social engagement, and emotion regulation rather than through entertainment value alone.

Emotional eating in response to emotions, such as stress, sadness, or boredom, is often used as a coping mechanism to regulate mood [8,12]. It has been associated with unhealthy dietary habits, overeating, and even overweight and obesity [13]. Research suggests that visual cues and emotional engagement with food-related media can stimulate appetite and influence eating behaviors [7,8,9]. Watching Mukbang could trigger cravings, normalize excessive food consumption, or provide emotional comfort, especially for individuals prone to emotional eating [14]. These include, but are not limited to, those who are stressed, depressed, lonely, and angry, such as adolescents and students [11,12,13]. Despite students being at risk of Mukbang addiction and its associations with eating behaviors due to highly stressful academic environments and high internet usage, few studies have been conducted in this population [8,14,15,16]. Saudi Arabia differs from the settings in which Mukbang has been studied. Food plays a central role in Saudi social life, where hospitality is expressed through generous portions and shared meals. These cultural practices differ from those of the East Asian and Western populations in which most Mukbang research has been conducted [17]. Saudi Arabia is also one of the most digitally connected countries. By the end of 2025, internet penetration reached 99% of the population, and social media user identities exceeded 38 million, equivalent to more than the total population [18]. At the same time, overweight, obesity, and emotional eating are common among Saudi young adults [13,19]. Findings from other populations should therefore not be assumed to apply directly to the Saudi context. Moreover, there is limited literature on Mukbang viewing in this context, as only one study has examined the association between Mukbang viewing and eating behaviors [20]. This study focused only on adults rather than students, indicating a lack of research on this population. Cultural practices also shape eating habits in this context [17].

Epidemiological evidence on Mukbang viewing is limited, but the available studies consistently report that the behavior is common and associated with unhealthy eating patterns. Reported prevalence ranges from 60.5% among Iranian female students [21] to 68.5% among Egyptian nursing students [14], while one U.S. study found that approximately one-third of adult viewers watched Mukbang daily or almost daily [7]. Mukbang viewing has also been associated with external eating [21], disordered eating symptoms [7], and, among adolescents, a dose-dependent increase in the odds of obesity [22]. Frequent television viewing combined with habitual snacking has been associated with an increased risk of obesity, metabolic syndrome, and insulin resistance in adults [23]. Despite these findings, no studies have examined Mukbang viewing among individuals with obesity or metabolic disorders, nor have they investigated its relationship with objective metabolic outcomes or focused on university students. The present study addresses these gaps by examining the relationship between Mukbang addiction, BMI, and emotional eating in a university student population.

University students make more of their own food choices than before, eat on irregular schedules, and manage limited budgets. They also experience academic stress, sleep disruption, and anxiety, all recognized triggers of emotional eating [12,13,24]. Their media habits may increase vulnerability, as late-night study and high smartphone use make solitary viewing more common, and eating while watching screens has been associated with poorer dietary quality among students [15]. In Saudi Arabia, students are also moving away from traditional family mealtimes toward eating alone or on campus, so the sense of companionship that Mukbang provides may be particularly appealing. Emotional eating has been documented among Saudi university students [19,25], yet its relationship with Mukbang engagement remains unexplored.

The present study focused on Mukbang addiction rather than viewing frequency because these concepts reflect different patterns of engagement. Viewing frequency reflects only how often an individual watches Mukbang videos, whereas addiction reflects the psychological significance of the behavior. Most viewers who watch Mukbang as a form of entertainment may not experience negative consequences. In contrast, problematic use is characterized by excessive viewing that begins to interfere with daily life, such as difficulty stopping, disrupted sleep or study, or changes in eating behavior [26]. Mukbang addiction represents the most severe level of engagement and includes features such as salience, mood modification, tolerance, withdrawal, conflict, and relapse, which are assessed by the Mukbang Addiction Scale (MAS) [27]. Unlike viewing frequency, addiction is closely linked to emotion regulation and impaired behavioral control, processes that also underlie emotional eating. Therefore, emotional eating was expected to be more strongly associated with Mukbang addiction than with viewing frequency.

Accordingly, this study aims to investigate the relationship between Mukbang addiction and emotional eating among university students in Saudi Arabia. Because both constructs involve affect regulation and reduced control over an appetitive behavior [28], higher MAS scores were hypothesized to accompany higher EEQ scores. BMI was examined as a secondary variable, since any pathway linking Mukbang engagement to intake should also register in body weight, and sociodemographic characteristics were examined by age, gender, education, marital status, occupation, or income. A cross-sectional design was acceptable since Mukbang exposure occurs naturally and without researcher interference. Furthermore, research into the incidence of Mukbang addiction and its associated determinants among Saudi university students is lacking.

Specifically, this study assessed the association between the addiction to watching Mukbang videos and the level of emotional eating among university students. Understanding this relationship may offer insights into how digital media consumption relates to emotional eating, contributing to public health and psychological well-being. Therefore, this study expands the data on Mukbang addiction and its relationship with emotional eating among university students.

## 2. Materials and Methods

### 2.1. Study Design and Participants

The study was conducted via the Google Forms online platform (Google LLC, Menlo Park, CA, USA). The study population was university students in Saudi Arabia, defined as any student currently enrolled at any higher-education institution in the country, whether public or private, with no restriction by university, region, or field of study. Recruitment occurred online via social media such as WhatsApp and Telegram, as well as university mailing lists. The sample size was calculated using Raosoft (Raosoft Inc., Seattle, WA, USA). The calculation was based on an estimated population size of approximately 500,000, a 95% confidence level, a 50% response distribution, and a 5% margin of error; a minimum sample size of 385 was required to meet the study objectives (*n* = 385). After excluding incomplete questionnaires, a total of 551 participants were included in the final analysis. Data collection took place between November 2025 and April 2026. Convenience sampling was used. The study sample comprised university students enrolled across Saudi Arabia. Students were included in the study if they (a) were 18 years of age or older, (b) were currently enrolled at any university in Saudi Arabia, and (c) were willing to participate in the study. The study was conducted in accordance with the Declaration of Helsinki and was approved by the Institutional Review Board (or Ethics Committee) of Qassim University (No. 25-13-01, issued on 16 November 2025). Before completing the survey, the participants read and provided informed consent. The purpose and objectives of the study, as well as any potential side effects, were explained. Participants were informed that their participation was voluntary and that they could withdraw at any time.

### 2.2. Questionnaire

A self-administered questionnaire was used. Experts in related fields reviewed the questionnaire’s scientific value in data collection. The questionnaire contains four sections: I. the aims and consent form; II. demographic information including age, gender, nationality, marital status, area of residence, education, income, occupation, suffering from disease, and participants provide their weight in kg and height in cm, which were used to calculate their Body Mass Index (BMI) (kg/m^2^) [29]; III. the Emotional Eating Questionnaire; and IV. the Mukbang Addiction Scale.

The Arabic version of the MAS was used to assess Mukbang Addiction [27], the original scale developed by Kircaburun et al. [26]. This scale consists of 6 items on a 5-point Likert scale, ranging from 1 (very rarely) to 5 (very often), with total scores ranging from 6 to 30. Higher scores indicate greater levels of Mukbang addiction. Participants were classified into low and high MA groups based on the sample mean and standard deviation of the total MAS score. This classification was specific to the current study and was not based on established cutoff values. The internal consistency of the MAS in the current study was (Cronbach’s α = 0.90).

The EEQ Arabic version was used to evaluate Emotional Eating Symptoms [19]. The questionnaire consists of ten items. The scoring is as follows: never = 0, sometimes = 1, generally = 2, and always = 3. Each response was assigned a score of 0–3, and the scores for all items were summed. The scoring interpretation is as follows: non-emotional eater (score 0–5); low emotional eater (score 6–10); emotional eater (score 11–20); and very emotional eater (score 21–30) [30]. The EEQ demonstrated good internal consistency (Cronbach’s α = 0.870) in the current study.

### 2.3. Statistical Analysis

Data were collected via the Google Forms online platform and analyzed using SPSS (version 26.0; IBM, Armonk, NY, USA) and Excel (Version 2205). Descriptive statistics included frequencies and percentages to summarize the categorical variables, whereas continuous variables were presented as mean ± standard deviation (SD). Questionnaires with incomplete responses were excluded from the analysis. The total MAS score was calculated, and categories were defined based on the mean ± SD of the total score. The chi-square test was used to evaluate relationships between categorical variables. Multiple linear regression analysis was performed with the MAS total score as the dependent variable to examine its association with the independent variables. Regression coefficients (B), standard errors (SE), standardized beta coefficients (β), 95% confidence intervals (95% CI), and *p*-values were reported. Multicollinearity was assessed using tolerance and variance inflation factor (VIF). A *p*-value < 0.05 was considered statistically significant.

## 3. Results

### 3.1. General Characteristics of Participants

A total of 551 responses were received from university students who completed the survey. Most participants were aged 18–24 years (58.3%) and male (55.9%). In terms of nationality and marital status, the majority of participants were Saudis and unmarried, at 93.6% and 73.5%, respectively. Moreover, the majority of participants were undertaking a bachelor’s degree (76.8%), and most were students (55%), followed by 38.5% who were students and also working. Most participants had an income of 1000 SR or less (47%). The participants were from different regions of Saudi Arabia, with the majority of them residing in the Central region (49.2%) and the least in the Northern region (2.4%). In terms of health status, the majority of participants (88.2%) did not suffer from any diseases, including diabetes, anemia, asthma, hypertension, or cardiovascular disease. The largest proportion of participants were of normal weight (42.3%), and the average weight and BMI were 72.38 kg (SD = 21.54) and 26.0 kg/m^2^ (SD = 10.12), respectively. Table 1 shows the frequency of demographic characteristics of the study participants.

### 3.2. Association Between Variables According to EEQ and MAS

The association between demographic characteristics and EEQ and MAS scores was determined. There were no significant differences in EEQ and MAS scores among participants across most variables, including age, gender, education, and income (*p* > 0.05). However, in terms of marital status and occupation, there were significant differences in EEQ (marital status: *p* = 0.025; occupation: *p* = 0.037) but not in MAS (marital status: *p* = 0.71; occupation: *p* = 0.20). On the other hand, participants differed significantly in both EEQ and MAS scores by BMI (*p* < 0.05). Table 2 shows the results of the association between the demographic variables and EEQ and MAS.

**Figure 1:**
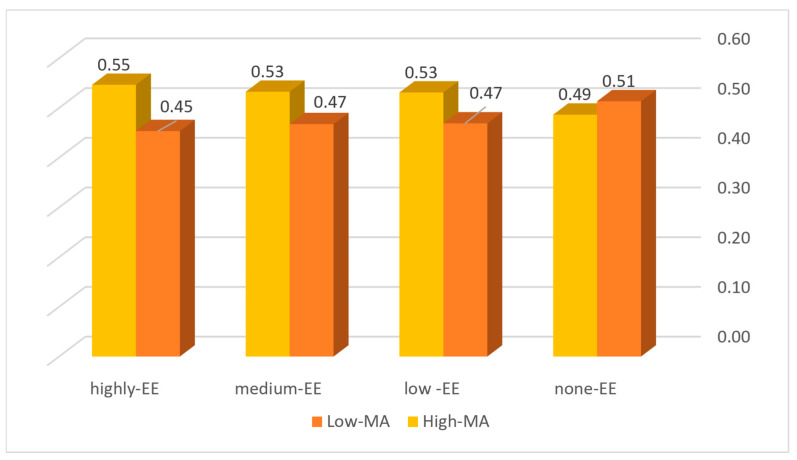
Association between Emotional Eating (EE) and Mukbang Addiction (MA) categories. Data show the distribution of Mukbang Addiction percentage across different Emotional Eating statuses. EE levels are categorized into None, Low, Medium, and High. MA levels are classified into Low and High Mukbang addiction.

The distribution of Mukbang addiction level across emotional eating status was also determined. Low, medium, and high emotional eating status had more participants with high Mukbang addiction levels than those with low Mukbang addiction levels. The high emotional eating status had the highest proportion of participants with a high level of Mukbang addiction (high MA—55%; low MA—45%). On the other hand, no emotional eating status was associated with more participants having low Mukbang addiction than those with high addiction (51% vs. 49%). Figure 1 shows the distribution of Mukbang addiction levels across emotional eating statuses.

### 3.3. Multiple Linear Regression Analysis of the Mukbang Addiction Scale

Multiple linear regression analysis was performed to identify associations between MA, demographic variables, and EES (Table 3). The results showed that MA was not significantly associated with any of the variables (*p* > 0.05). EEQ, in particular, showed a weak positive association with MA, but this association was not significant (B = 0.275, *p* = 0.249). Multicollinearity was assessed using VIF and tolerance statistics, and no evidence of multicollinearity was identified.

## 4. Discussion

To the best of our knowledge, no study has examined Mukbang addiction and its association with emotional eating among students in Saudi Arabia, despite the literature suggesting that problematic Mukbang watching is associated with dietary habits, including emotional eating behaviors [3,11,26,31]. Therefore, this study aimed to evaluate the association between Mukbang addiction and students’ emotional eating in Saudi Arabia. This is important considering the high level of vulnerability of this population group due to stressful academic environments, heightened anxiety, and limited budgets that may lead to Mukbang watching as a coping mechanism for these negative emotions [8]. Consequently, this study offers insights into the students’ emotional eating behaviors and informs targeted public health interventions.

Mukbang addiction did not differ across any of the sociodemographic characteristics examined, indicating that Mukbang addiction is not strongly influenced by demographic factors. Given the high level of internet and social media use in Saudi Arabia [18], Mukbang content is readily accessible to most students, which may explain the lack of demographic variation in viewing patterns. The absence of a gender difference is particularly notable, given that emotional eating is more frequently reported among women [8,9]. This finding suggests that, although susceptibility to emotional eating may vary by gender, exposure to Mukbang content is similar among male and female students. No age-related differences were identified, possibly reflecting the relatively narrow age range and the high level of digital media use among the sample. Similar to these findings, previous studies have also found no significant association between Mukbang watching and age [20,21] and education [21]. However, Alissa and Alhussain [20] showed that education was significantly associated with Mukbang watching, with those of higher educational level reporting less frequent viewing. This contradictory finding could be attributed to differences in participants: Alissa and Alhussain [20] sampled the general population, whereas the present study was conducted in a university setting. Age, gender, education, and income were not significantly associated with emotional eating behaviors. However, BMI was significantly associated with both Mukbang addiction and emotional eating, contradicting the findings of Anari and Eghtesadi [21] but supporting those of Jeong et al. [22], who showed a significant association between Mukbang viewing and BMI among adolescents. Mukbang addiction has been associated not only with disordered eating but also with poor eating habits, including highly processed foods [32], which are in turn associated with a higher risk of overweight and obesity [13].

BMI was the only variable significantly associated with Mukbang addiction in this study. Unlike emotional eating, which reflects eating in response to negative emotions, BMI is influenced by a wide range of long-term behavioral and lifestyle factors. Mukbang addiction may instead be associated with everyday habits, such as eating while watching screens, prolonged sedentary behavior, and repeated exposure to energy-dense foods, all of which have been linked to weight gain [7,15,32]. Alternatively, individuals with a higher BMI might engage more with food-related media because of greater interest in food or repeated exposure through personalized content recommendations. The relationship could also run in both directions, with Mukbang addiction influencing eating behaviors and higher body weight encouraging greater engagement with food-related content. However, because of the cross-sectional design, the temporal sequence of this association cannot be established. In addition, unmeasured factors, including dietary restraint, body-image concerns, physical activity, sleep, and overall screen time, may have influenced the observed association. Overall, the association between Mukbang addiction and BMI appears to reflect habitual eating and media-use behaviors rather than emotion-driven eating.

The results on the association between Mukbang addiction and emotional eating showed that although Mukbang addiction had a positive association with emotional eating, this association was weak and non-significant (B = 0.275, *p* = 0.249). The findings suggest that Mukbang addiction is not significantly associated with students’ emotional eating. Similar to these findings, Anari and Eghtesadi [21] found no significant relationship between the frequency of Mukbang watching and emotional eating behavior among Iranian female students. Alissa and Alhussain [20] also found no significant association between Mukbang watching and emotional eating behaviors. These could be attributed to this population watching Mukbang mainly for recreational purposes or for its perceived benefits, rather than as a coping strategy for negative emotions. For instance, some authors have suggested that if viewed appropriately, Mukbang can promote positive eating habits [33].

In contrast, some studies have reported a significant relationship between Mukbang addiction and emotional eating [16,31]. For instance, Bölükbaşı et al. [31] found that watching Mukbang was positively associated with emotional eating attitudes. Similarly, Elkin et al. [16] showed that Mukbang addiction was significantly associated with emotional eating. These associations between Mukbang addiction and emotional eating have been attributed to maladaptive changes in eating habits, including changing food preferences and promoting disordered eating [3,31].

In an earlier study of female students at Qassim University, Alharbi and Alharbi reported that emotional eating was associated with higher fat intake and higher educational level, and that fat consumption was associated with emotional eating, while no clear link was found with BMI [25]. In general, this study indicates that Mukbang addiction is not significantly associated with emotional eating among students. Moreover, it shows that sociodemographic variables are not associated with Mukbang addiction. However, it appears to be linked to BMI.

The cultural context of Saudi Arabia may help to explain why the present findings differ from those reported in East Asian and Western samples, where Mukbang engagement has been associated with altered eating attitudes and dietary habits [11,15,21,31]. Shared meals and generous hospitality are the norm in Saudi society [17]. Companionship at mealtimes is already available to most students, so the parasocial sense of “eating together” that motivates Mukbang engagement where solitary eating is common may be less salient here [5,6]. This would weaken the link between Mukbang addiction and emotional eating. Large quantities of food are also culturally familiar rather than novel, so exposure to Mukbang portions may carry less of the portion-normalizing influence proposed in other populations [3,11]. Viewing may therefore function mainly as entertainment or as a way to explore unfamiliar cuisines rather than as a strategy for regulating negative emotions [20,22], which is consistent with the absence of a significant association in this sample. Exposure nevertheless remains common, given the very high level of digital connectivity in Saudi Arabia [18]. The association observed here between BMI and both Mukbang addiction and emotional eating is in line with studies reporting a relationship between eating-broadcast viewing and body mass index [22,32], as well as with evidence that emotional eating is frequently reported among Saudi university students [19,25]. Because commensality, hospitality norms, and religious eating practices were not measured, these interpretations remain untested. Confirming them would require qualitative or cross-cultural research.

The current study has limitations, as it was conducted in a non-clinical setting. Therefore, studies should be conducted with a larger sample size of participants from different areas in the Kingdom of Saudi Arabia. To improve dietary intake in this young population, future interventions may take into account emotional eating and self-regulation of eating. Another limitation of this study is that it relied solely on participants’ self-reports, which are subject to bias; BMI was calculated from self-reported height and weight rather than from measured values and may therefore be misclassified. In addition, several variables likely to be relevant to the BMI association, including dieting history, restrained eating, weight and body-image concerns, physical activity, and total screen time, were not assessed, so residual confounding cannot be ruled out. Because the study did not use a longitudinal design, it did not follow the participants over an extended period. Most importantly, the cross-sectional design means that the associations reported here cannot establish causality or the temporal ordering of Mukbang engagement and emotional eating; longitudinal or experimental studies are required to determine whether either behavior precedes or influences the other. Finally, no biochemical or objective metabolic measures were obtained; lipid profile, glucose, insulin resistance, and body composition were outside the scope of this survey-based design, so these data cannot speak to whether Mukbang engagement is related to lipid accumulation or other metabolic outcomes. Future work should combine repeated measures of Mukbang engagement with measured anthropometry, dietary intake records, and metabolic biomarkers, ideally within longitudinal or experimental designs, to establish whether the behavioral associations reported here translate into measurable metabolic risk.

## 5. Conclusions

In this cross-sectional sample of university students in Saudi Arabia, Mukbang addiction and emotional eating were not significantly associated, whereas BMI was significantly associated with both. Longitudinal research is needed to clarify the temporal relationships between Mukbang engagement and emotional eating and to identify potential mediators. Further research should also examine both the potential benefits and risks of Mukbang content in relation to students’ eating behaviors.

## Figures and Tables

**Table 1:** The frequency of participant characteristics.

Variables	Items	Total Sample	
		N	%
Age	18–24	321	58.3
	25–34	133	24.1
	35–44	74	13.4
	45 and more	23	4.2
Gender	Female	242	43.9
	Male	308	55.9
Nationality	Saudi	517	93.8
	non-Saudi	34	6.2
Marital status	Married	146	26.5
	Unmarried	405	73.5
Area of residence	Central Region	271	49.2
	Eastern Region	30	5.4
	Western region	83	15.1
	Northern region	13	2.4
	Southern region	154	27.9
Education	Diploma or less	20	3.6
	Bachelor	423	76.8
	Postgraduate	108	19.6
Occupation	Work and student	212	38.5
	Freelancer and student	36	6.5
	Students	303	55.0
Incomes	1000 or less	259	47.0
	2000–5000	108	19.6
	more than 5000	184	33.4
Suffering from a disease	Diabetes	11	2.0
	Anemia	38	6.9
	Asthma	8	1.5
	Hypertension	6	1.1
	Cardiovascular diseases	2	0.4
	No one suffers from disease	486	88.2
BMI categories	Underweight	53	9.6
	Normal weight	233	42.3
	Overweight	154	27.9
	Obesity	111	20.1
Weight	Mean ± SD	72.383 ± 21.54	
BMI		25.998 ± 10.12	

Data presented as n (%) for categorical variables. Mean ± (SD) Standard Deviation.

**Table 2:** Association between variables according to EEQ and MAS.

Variables	Items	Totals	EEQ				*p*-Value	MAS		*p*-Value
			None	Low	Medium	High		Low	High	
Age	18–24	321	46 (14.3)	95 (29.6)	139 (43.3)	41 (12.8)	0.082	139 (43.3)	182 (56.7)	0.63
	25–34	133	14 (10.0)	29 (21.8)	68 (51.1)	22 (16.5)		76 (57.1)	57 (42.9)	
	35–44	74	9 (12.2)	16 (21.6)	29 (39.2)	20 (27)		34 (45.9)	40 (54.1)	
	45 and more	23	5 (21.7)	5 (21.7)	10 (43.5)	3 (13)		11 (47.8)	12 (52.2)	
Gender	Female	242	45 (14.6)	84 (27.2)	135 (43.7)	45 (14.6)	0.680	152 (44.6)	157 (55.4)	0.29
	Male	308	29 (39.2)	61 (42.1)	111 (45.9)	41 (16.9)		108 (49.2)	134 (50.8)	
Education	Diploma or less	20	2 (10)	3 (15)	11(55)	4 (20)	0.109	5 (25)	15 (75)	0.054
	Bachelor	423	65 (15.4)	115 (27.2)	184 (43.5)	59 (23.9)		197 (46.6)	226 (53.4)	
	Postgraduate	108	7 (6.5)	27 (25)	51 (47.2)	23 (21.3)		58 (47.2)	50 (46.3)	
Marital status	Married	146	15 (10.3)	29 (19.9)	71 (48.6)	31 (21.2)	0.025	67 (45.9)	79 (54.1)	0.71
	Unmarried	405	59 (14.6)	116 (28.6)	175 (43.2)	55 (13.6)		193 (47.7)	212 (52.3)	
Occupation	work and student	212	29 (13.7)	46 (21.7)	95 (44.8)	42 (19.8)	0.037	108 (50.9)	104 (49.1)	0.20
	freelancer and student	36	4 (11.1)	9 (25)	13 (36.1)	10 (27.8)		13 (36.1)	23 (63.9)	
	student	303	41 (13.5)	90 (29.7)	138 (45.5)	34 (11.2)		139 (45.9)	160 (54.1)	
Incomes	1000 or less	259	32 (12.4)	70 (27)	121 (46.7)	36 (13.9)	0.734	115 (44.4)	144 (55.6)	0.43
	2000–5000	108	15 (13.9)	32 (29.6)	45 (41.7)	16 (14.8)		52 (48.1)	56 (51.9)	
	more than 5000	184	27 (14.7)	43 (23.4)	80 (43.5)	34 (18.5)		93 (50.5)	91 (49.5)	
BMI	Underweight	53	14 (26.4)	22 (41.5)	16 (30.2)	1 (1.9)	0.00	21 (39.6)	32 (60.4)	0.023
	Normal weight	233	40 (17.2)	68 (29.2)	97 (41.6)	28 (12)		114 (48.9)	119 (51.1)	
	Overweight	154	10 (6.5)	35 (22.7)	82 (53.2)	27 (17.5)		84 (54.5)	70 (45.5)	
	Obesity	111	10 (9)	20 (18)	51 (45.9)	30 (27)		41 (36.9)	70 (63.1)	

Data presented as n (%) for categorical variables; *p*-value < 0.05 considered significant.

**Table 3:** Multiple linear regression analysis.

Variables	B ± SE	Std. Beta	95.0% Confidence Interval for B		*p*-Value
			Lower Bound	Upper Bound	
Age	−0.111 ± 0.388	−0.020	−0.873	0.650	0.774
Gender	0.190 ± 0.441	0.020	−0.677	1.056	0.667
Education	−0.869 ± 0.513	−0.083	−1.878	0.139	0.091
Marital status	0.102 ± 0.604	0.009	−1.085	1.289	0.866
Occupational	0.225 ± 0.430	0.045	−0.620	1.070	0.601
Income	0.459 ± 0.466	0.085	−0.456	1.374	0.325
EEQ categories	0.275 ± 0.239	0.052	−0.193	0.744	0.249
BMI categories	−0.201 ± 0.255	−0.039	−0.703	0.301	0.431

Data presented as B ± SE from multiple linear regression. The dependent variable was the total MAS score. *p* < 0.05 was considered statistically significant.

## Data Availability

The raw data supporting the conclusions of this article will be made available by the authors on request.

## Abbreviations

The following abbreviations are used in this manuscript:

MAS	Mukbang Addiction Scale
EEQ	Emotional Eating Questionnaire
MA	Mukbang Addiction
EE	Emotional Eating
BMI	Body Mass Index
